# A comparative study of the use of extended reality simulation in neonatal resuscitation training

**DOI:** 10.1186/s41077-025-00344-4

**Published:** 2025-03-25

**Authors:** Mustafa Yalin Aydin, Vernon Curran, Susan White, Lourdes Peña-Castillo, Oscar Meruvia-Pastor

**Affiliations:** 1 Department of Computer Science, Faculty of Science, Memorial University of Newfoundland, St. John’s, NL Canada; 2 Division of Population Health and Applied Health Sciences, Faculty of Medicine, Memorial University of Newfoundland, Room H2982, Health Sciences Centre, St. John’s, NL Canada; 3 Perinatal Program NL, Newfoundland & Labrador Health Services, Janeway Pediatric Research Unit, Janeway Hostel, Room 413, 300 Prince Philip Drive, St. John’s, NL Canada; 4 Departments of Computer Science and Biology, Faculty of Science, Memorial University of Newfoundland, St. John’s, NL Canada

**Keywords:** Virtual reality, Neonatal Resuscitation Program, Education, Randomized controlled trial

## Abstract

**Background:**

360° video and virtual reality (VR) simulation may offer innovative opportunities as portable simulation-based technologies to enhance Neonatal Resuscitation Program (NRP) training, updates, and refreshers. The purpose of this study was to compare the use of 360° video with VR simulation in NRP training and the effect on NRP learning outcomes.

**Methods:**

Thirty (*N* = 30) NRP providers were randomly assigned to either VR simulation or 360° video study groups (*n* = 15 each) with pre and posttests of confidence, posttests of user satisfaction, usefulness, presence, and simulator sickness, and a performance demonstration of positive pressure ventilation (PPV) on a manikin-simulator. Participants were then exposed to the other condition and again post-tested.

**Results:**

Both systems were positively viewed. However, participants reported significantly higher perceptions of usefulness in enhancing learning and increased sense of presence with the VR simulation. VR simulation participants gained more confidence in certain NRP skills, such as proper mask placement (adjusted *p*-value 0.038) and newborn response evaluation (adjusted *p*-value 0.017). A blinded assessment of PPV skills showed participants exposed to VR performed significantly better in providing effective PPV (adjusted *p*-value 0.005).

**Conclusions:**

NRP providers found both systems useful; however, VR simulation was more helpful in improving learning performance and enhancing learning. Participants reported an increased feeling of presence and confidence in certain areas with VR and performed better on a crucial NRP skill, providing effective PPV. VR technologies may offer an alternative modality for increasing access to standardized and portable refresher learning opportunities on NRP.

## Background

An estimated 2.4 million newborns die in the first 28 days of life, with up to 10% needing help to begin breathing at birth and 1% requiring resuscitation to restore cardiorespiratory function [1]. Neonatal resuscitation training is crucial for enhancing positive patient outcomes of newborns. The Neonatal Resuscitation Program (NRP) was introduced in 1987 by the American Heart Association and the American Academy of Pediatrics to train healthcare providers on the resuscitation of the newborn [2–6]. To maintain current provider status in Canada, the Canadian Pediatric Society requires practitioners responsible for the care of the newborn to complete an online examination and an in-person NRP provider course every 2 years.

An essential concern for neonatal resuscitation is knowledge and skill retention following training, as resuscitation ability has been shown to degrade rapidly, with some studies suggesting deterioration within weeks [2, 7–9]. Booster/refresher sessions involving “mock codes” and simulation-based education using manikin-simulators can improve knowledge retention and skill updating [2, 9–11]. Unfortunately, access to manikin-simulation equipment and timely booster sessions may be challenging in rural contexts, limited by equipment and travel costs or lack of availability and time [2, 11, 12].

Extended reality (XR) has emerged as a new method of creating simulated learning experiences that are more cost-effective than traditional simulation modalities [13–15]. XR is an umbrella term referring to all immersive technologies typically supported by the use of head-mounted displays (HMDs), including virtual reality (VR), augmented reality, and mixed reality [14, 16, 17]. These technologies offer greater portability with no manikin components to transport, greater content standardization, replicability of experiences, and no consumable parts requiring regular replacement [12, 14, 15, 18, 19]. Immersive XR also has the potential to enhance learner engagement, increase satisfaction, and foster greater contextualization for clinical sciences learners [14, 18, 20–22].

VR simulation and 360° video are two common extended reality modalities in medical education [14]. VR refers to a 3D simulation that allows real-time interaction and immersion in virtual environments that can produce visual, auditory, and haptic sensory experiences [14]. These virtual environments are visualized using an HMD (VR headset), allowing users to interact with virtual objects [23]. Hands, gloves, joysticks, and VR controllers are sometimes used to facilitate interaction with the virtual environment [24]. 360° video encompasses video recordings of real-world scenes that produce a totally immersive, 3D experience using a VR headset [14, 25]. These 360° cinematic images are updated in real-time as the person looks around and switches from one scene to the next [14]. However, unlike VR and augmented reality, users cannot move around or interact directly with objects in the scene [14].

Contextual or “authentic” learning involves knowledge and skill acquisition in contexts that reflect how these abilities may be applied in real life [26]. It encompasses using instructional methods that replicate, simulate, or immerse learners in an environment that reflects the practical circumstances between structured learning activities and the context in which they may be expected to perform. Simulation-based education using XR systems has been identified as a highly effective method that immerses learners in realistic situations created within a virtual space replicating the authentic environment [26].

Immersion and presence are two critical features of VR technology. Immersion refers to a psychological state in which one feels as though they are interacting directly within a simulated environment [27]. Greater immersion contributes to an increased level of presence, which has been described as a subjective experience of feeling as though one is in a different place than where one is physically situated [27]. Creating a sense of presence through immersion is one goal of using immersive VR in education and training and a key feature conducive to learning [2, 18]. Nonetheless, some studies have demonstrated that head-mounted displays can cause side effects such as nausea, headaches, and vertigo, also called simulation-induced sickness. However, such effects have become rarer with state-of-the-art computer systems, graphics cards, and headsets. Yet, evaluating the side effects of extended reality use with newer headsets, systems, and novel simulations is important to examine the extent to which these effects are still a relevant limitation for most users [14].

Several studies have reported using XR in neonatal resuscitation training; however, learning effects remain unexamined. Ghoman et al. [4] have described the Electronic Helping Babies Breathe VR game that can be accessed using low-cost VR devices such as Google Cardboard and enables users to observe neonatal resuscitation procedures through their smartphones. The University of Newcastle, Australia has also developed an immersive VR application to teach midwifery students neonatal resuscitation skills [28]. Curran et al. [2] explored the use of 360° neonatal resuscitation training videos using VR headsets and found a high level of acceptance amongst NRP providers. However, comparative studies examining different XR systems and their impact on neonatal resuscitation learning outcomes are limited. Yang and Oh [29] reported a study comparing a neonatal resuscitation gamification program using VR with high-fidelity simulation and online lectures and a control group that was just receiving online lectures. The VR group participants demonstrated significantly higher problem-solving ability and self-confidence than the simulation and online lecture groups. In other resuscitation fields, Kuyt et al. [30] scoping review found an increasing use of VR and augmented reality in cardiopulmonary resuscitation education and training for healthcare professionals and positive effects on practical skills learning comparable with traditional classroom methods. Such technologies were also well received by instructors and healthcare providers, particularly among younger generations [30].

This study examines and compares 360° video with VR simulation in neonatal resuscitation booster training and the effect on user satisfaction, confidence, simulator sickness, and ability to perform effective positive pressure ventilation (PPV).

## Methods

We conducted a randomized pretest–posttest crossover study. Thirty (*N* = 30) current NRP providers were recruited between August and December of 2021 with the assistance of clinical educators through email and posters displayed in the healthcare facility, Neonatal Intensive Care Unit, and the affiliated medical school. An online calendar was made available for self-registration and participants were requested to register for an available session based on their availability. Study participants were assigned to one of two study groups by random sampling without replacement to ensure an equal number of participants (*N* = 15) in each group.

The study was conducted in the Office of Professional & Educational Development facilities at Memorial University of Newfoundland. The experimental conditions included the following: Group A, VR-NRP simulation prototype produced explicitly for this study; and Group B, 360° NRP training video (a.k.a. 360° video), produced initially by Curran et al. [2] (https://youtu.be/wW0R57-4_KQ). Both conditions were facilitated by a technical operator with expertise in computer simulation, who provided the NRP provider with an HMD containing either the VR-NRP simulation or the 360° video and offered troubleshooting. Once set up, the NRP provider worked through the condition independently until finished, at which point the operator assisted with removing the HMD and arranged the setup for the other condition. The duration of the study per participant was approximately 1 h. The NRP algorithms and resources were the same between the VR and the 360° video conditions.

### VR simulation prototype

**Fig. 1:**
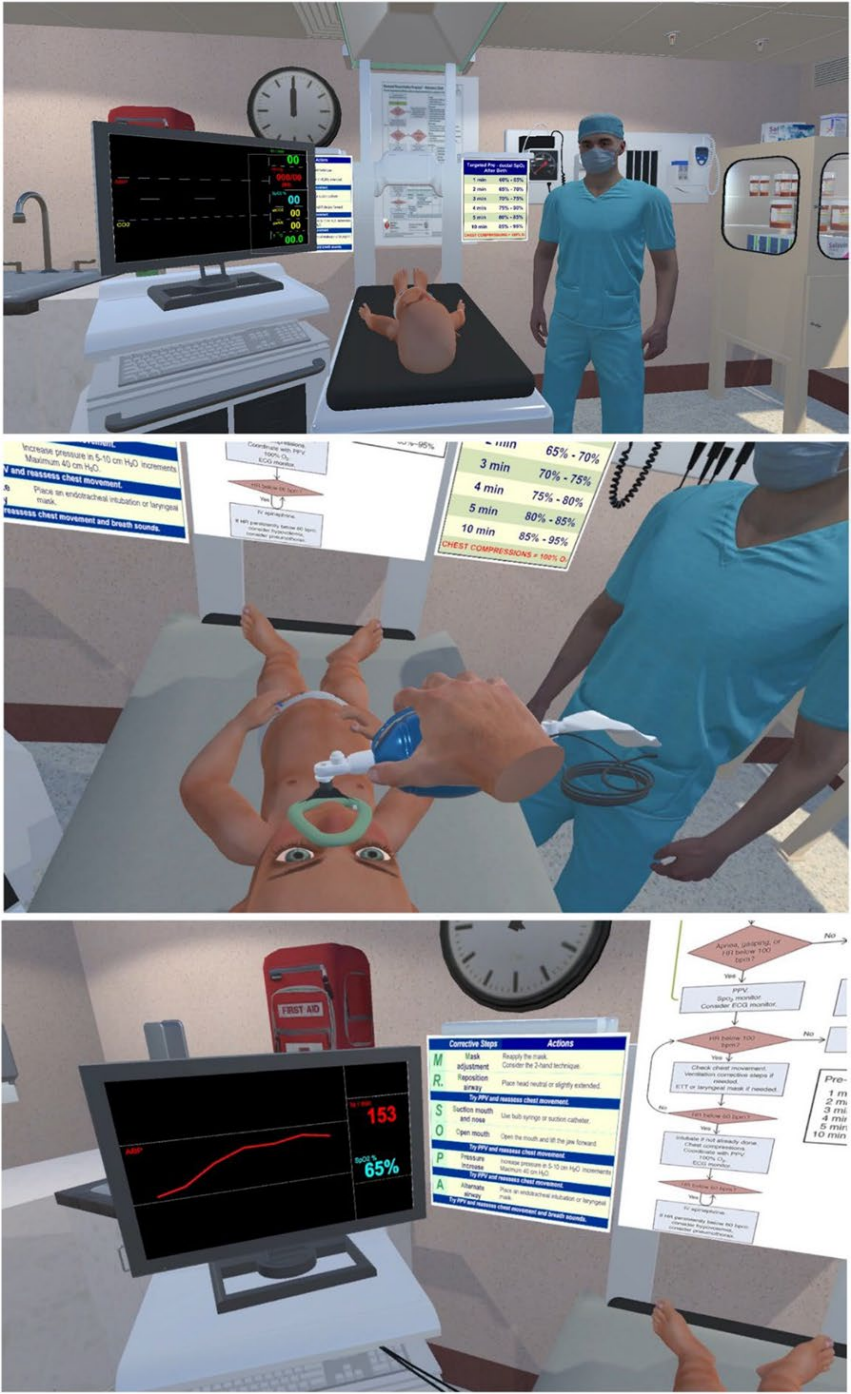
Sample images from the simulation. Top: View of the VR simulation room from the user’s perspective. Center: The user holds the mask to the baby’s face, holds the bag valve mask with right hand, and does PPV. Bottom: The monitor is used to reflect the information it receives from the ECGs attached to the baby’s chest. It is on the left side of the user’s viewpoint and the values change every second

The VR simulation prototype (https://youtu.be/r40twTjuwTE) was developed at Memorial University by MYA (first author) using the Unity 3D game engine (version 2020.02f) and its integrated development environment (https://unity.com/products/unity-engine). The prototype was developed in an Omen 15 Windows 10 Laptop with 16 GB of RAM and an NVIDIA RTX 2060 GPU with 1 GB RAM. An HTC VIVE Pro headset was utilized with the SteamVR plugin for controller interactions and VR environment management. Figure 1 depicts the VR simulation environment.

The provider first enters a triage room where a VR menu gives options between a tutorial, the NRP simulation, and the exit. The tutorial explains how to use the VR controllers to interact with the virtual objects in the simulation. The VR NRP simulation follows the recommendations outlined in the 7th edition of the NRP book [3]. The objective of the simulation is to allow the NRP provider to resuscitate a virtual newborn on a warmer. The procedure is executed interactively, where the user collaborates with a virtual assistant, who provides information about the baby, monitors the newborn’s vitals, and provides instructions on the steps to follow. The provider places the mask on the newborn and applies positive pressure ventilation by pressing the trigger button on the VR controller. The simulation adapts to the pace of the provider, allowing time to complete the steps and repeating prompts if necessary. A more detailed description of the prototype, which includes descriptions of the training prompts, objectives, user navigation, expected behaviors, types of interactions, interface for interactions, environment, and embedded feedback is presented here: 10.48550/arXiv.2406.15598. 

### 360° NRP training video

**Fig. 2:**
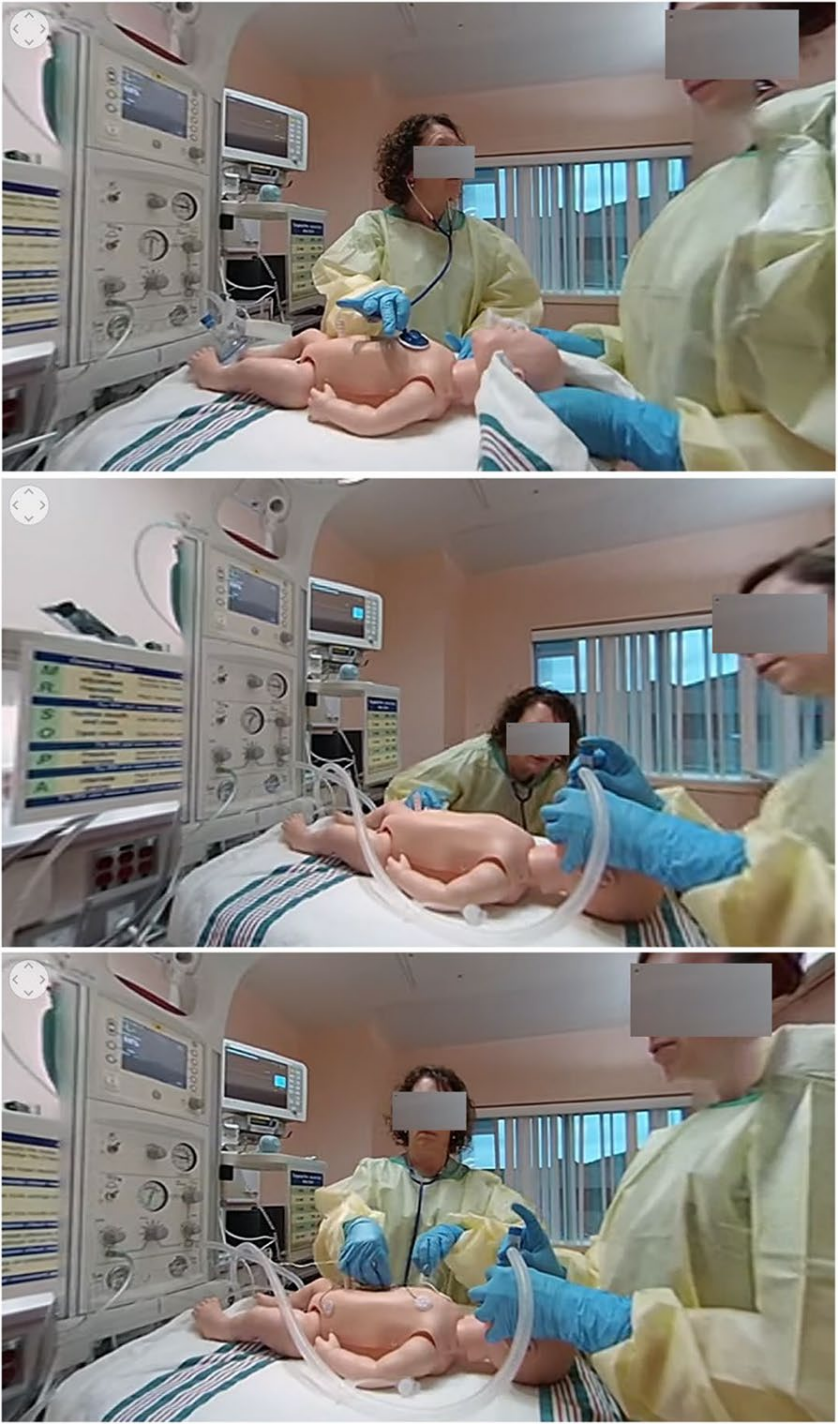
Sample images from the 360° video. Top: NRP provider checking the newborn’s heart rate. Center: Provider checking for the newborn's chest to rise. Bottom: Provider placing electrodes to monitor newborn’s vitals

The 360° NRP training video condition was developed at Memorial University and is detailed in Curran et al. [2]. In this condition, participants viewed the video using the Oculus GO. The scene starts with the provider looking at a newborn manikin lying on a warmer and being assessed and resuscitated by the demonstrators in the video from a 3rd person perspective. The objective is to immerse the user in a realistic scenario of the NRP procedure. The participant cannot walk around, interact with the environment, or participate in the procedure, but can turn their gaze around 360° within the room. Screenshots of this view are provided in Fig. 2.

### Experimental design

**Fig. 3:**
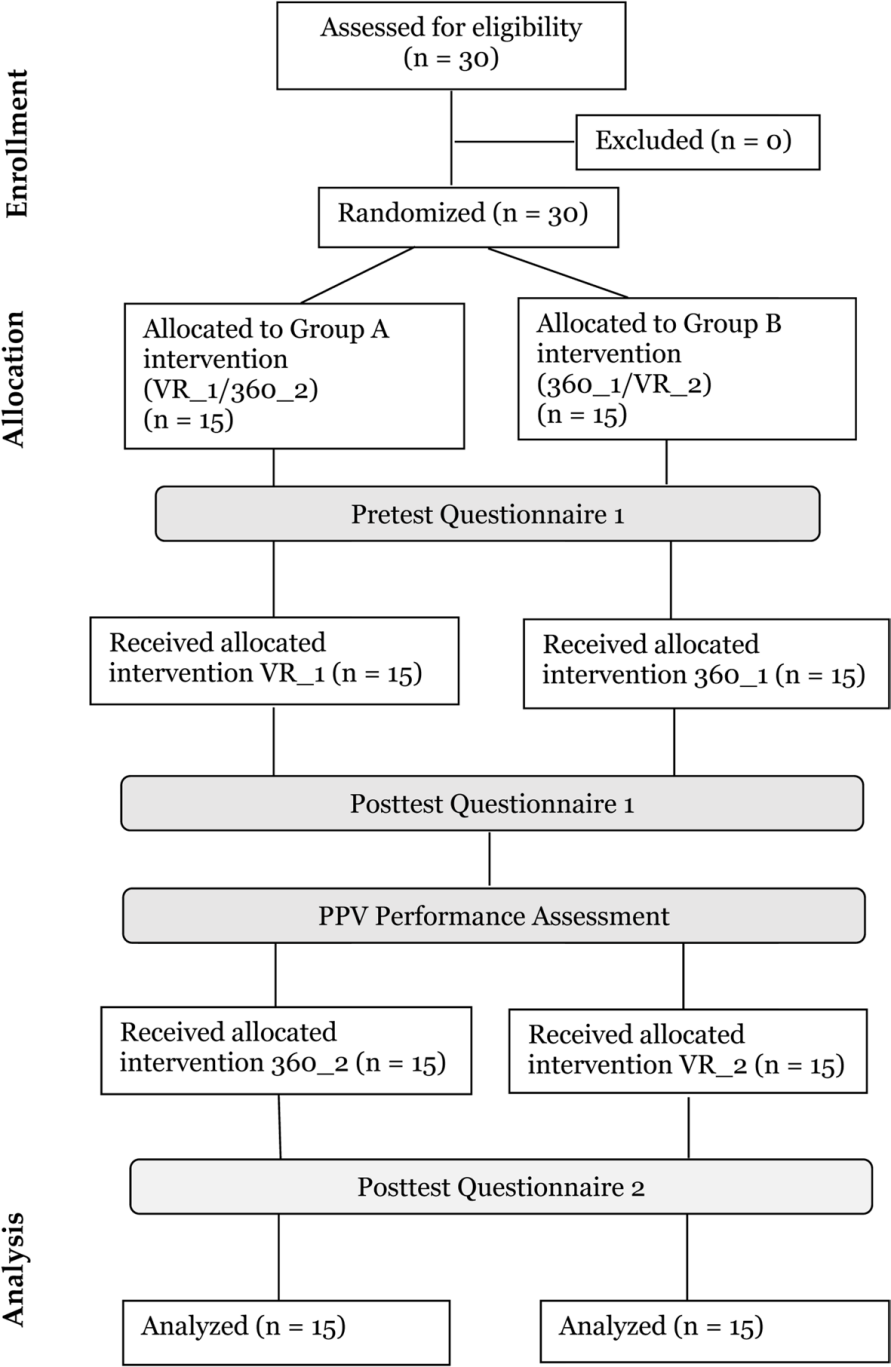
CONSORT diagram for randomized pretest–posttest crossover study of the effect of extended reality (XR) on Neonatal Resuscitation Program (NRP) learning outcomes

The crossover design allowed participants to try both experimental conditions during the same session, although in a different order (Fig. 3). Group A was exposed to the VR simulation first (VR_1) and the 360° NRP training video second (360_2), whereas Group B experienced the reverse order, 360° NRP training video first (360_1) and then VR simulation (VR_2). Before exposure, a paper-based pretest was completed by participants, which included a demographic questionnaire adapted from Curran, Fleet, and Greene [31] (e.g., gender, health profession, years in practice, years as NRP provider, and NRP experience) and a 15-item neonatal resuscitation confidence questionnaire developed by Curran et al. [32]. This questionnaire was validated initially against the Canadian adaptation of the Basic Megacode Assessment Form [32]. Respondents were asked to rate their confidence level using a scale of “0 = cannot do at all to 100 = highly certain can do”. Following the first condition (VR_1 or 360_1), participants were asked to complete a paper-based posttest questionnaire that evaluated user satisfaction, confidence, sense of presence, and simulator sickness. User satisfaction (in terms of usefulness and ease of use) was measured using two 6-item Likert-scale survey question sets (1 = extremely unlikely to 7 = extremely likely) adopted from Chávez et al. [33].

Presence was evaluated using an adapted version of the 32-item Presence Questionnaire (PQ) [27]. The PQ uses a 7-point scale format addressing four main conceptual factors that influence involvement and immersion in the learning experience. Some questions used in the PQ were removed for the 360° video condition as they were not applicable, given constraints including the lack of user mobility, control of virtual objects, and interaction with the scene. Simulator sickness was assessed using a 4-point Simulator Sickness Questionnaire (SSQ) (1 = none, 2 = slight, 3 = moderate, and = severe) adopted from Kennedy et al. [34] that measures self-reported simulation-induced sickness across 16 symptoms and was originally validated by testing with subjects using flight simulators [35].

Upon completing the first experimental condition (VR_1 or 360_1), participants were asked to perform the positive pressure ventilation resuscitation procedure on a newborn manikin (Gaumard HAL low fidelity neonate) using a flow-inflating or self-inflating device, based on which device they used in their clinical area while being recorded. All participants were exposed to the same scenario, assistant, and environment and were read the same simulation brief and patient details from a standard script outlining information regarding pregnancy and delivery up to and including completion of the “initial steps of resuscitation”. The evaluation criteria began at the same point, with the participant being required to respond to a newborn with no respiratory effort and an initial heart rate of 50 beats per minute.

The video recordings were distributed among 3 experienced instructors for scoring (10 each, 5 VR and 5 360°), blinded to which condition the participant had been exposed to. A standard NRP observation checklist, adapted from the Canadian version of the NRP 7th edition Integrated Skills Station Assessment, was used to assess the participants’ ability to recognize the need for PPV, initiate PPV, assess effectiveness, use the recommended steps to fix ineffective ventilation, provide effective PPV for 30 s, and reevaluate the newborn. The instructors evaluated participants’ performance on 10 criteria for a maximum score of 20. Items were rated on a scale of “0 = not done, 1 = done incorrectly, incompletely or out of order, and 2 = done correctly in order”.

After participants experienced the second condition (VR_2 or 360_2), they were asked to complete another questionnaire (posttest 2) that again measured user satisfaction, sense of presence, and degree of simulator sickness. Survey data was anonymized and recorded in a password-protected spreadsheet. Ethical approval was received from Memorial University’s Interdisciplinary Committee on Ethics in Human Research, and participants were required to complete an ethics consent form.

### Data analysis

To test whether the composition of study groups for gender, professional role, and NRP experience was like that obtained by random participant group allocation, we performed Pearson’s chi-square tests with *p*-values calculated by Monte Carlo simulation with 10,000 replicates. To test whether participants’ responses to Likert-scale questions differed among study groups, we performed pair-wise Wilcoxon (also known as Mann–Whitney) tests with false-discovery rate correction for multiple testing. Gain in confidence level per study group was assessed by comparing the difference between the participants’ pre- and post-test confidence levels after the first condition. ANOVA analyses were done to evaluate the effect of condition, order, and the interaction between condition and order in the mean scores. All statistical data analyses were done in R (version 4.1.3). Plots were created using the R data visualization package ggplot2 (version 3.4.0).

## Results

Fifty percent (50%) of study participants were Registered Nurses (15/30), while 16% were pediatric residents (5/30). Other participants included 1 Licensed Practical Nurse, 2 Pediatricians, 2 Clinical Associates, 2 Obstetric residents, 1 Respiratory Therapist, 1 Medical student, and 1 Respiratory Therapy student. Most participants were female (93%, *n* = 28); had completed their last NRP provider course within the past year (53%, *n* = 16); and reported between 0 and 5 years of experience in practice (73%, *n* = 22). There were no statistically significant differences between study group composition based on gender (*p*-value = 0.476), professional roles (*p*-value = 0.934), years in practice (*p*-value = 0.337), years as an NRP provider (*p*-value = 1), nor last NRP provider course (*p*-value = 0.197). There was also no statistical difference in participants’ pre-confidence scores before completing either 360^○^ video or VR simulation (*p*-values range across the 15 questions was [0.24, 1]). In sum, both study groups were similar in terms of demographic composition and participants’ pre-confidence.

**Fig. 4:**
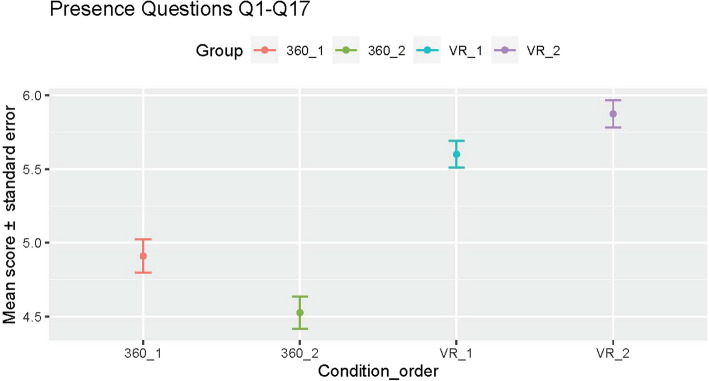
Mean score and standard error for the sense of presence when grouping by condition and order

A comparison of the pre- and post-confidence scores for each group showed participants in the VR simulation study group (VR-1 vs 360_1) reported gaining higher confidence in Q3: “demonstrating correct mask placement” (adjusted *p*-value 0.04) and Q10: “re-evaluating newborn response after 30 s of effective PPV (HR and spontaneous respirations)” (adjusted *p*-value 0.02). When presence was compared between the 360° video and VR groups, 14 out of 17 questions showed significantly higher scores amongst the VR group (with the corresponding *p*-values ranging from 8.43 × 10^−7^ to 0.04), suggesting higher perception of presence in the VR simulation (mean presence score for questions 1 to 17, was 4.7 ± 1.78 for 360° video and 5.7 ± 1.47 for VR). The mean score for questions 18 to 32 in the VR condition was 4.6 ± 1.85, suggesting a positive perception of VR-specific features. Participants generally reported a higher perception of presence in the VR simulation than in the 360° video (regardless of the order); however, this perception increased for VR_2 and decreased for 360_2 (Fig. 4). The results from an ANOVA analysis suggest that the most significant factor in the participants’ score is the condition (F statistic 152.7, *p*-value < 2 × 10^−16^), the order is not a significant factor (F statistic 0.44, *p*-value 0.51); and there is a significant interaction effect between condition and order (F statistic 15.9, *p*-value 7.0 × 10^−5^).

**Fig. 5:**
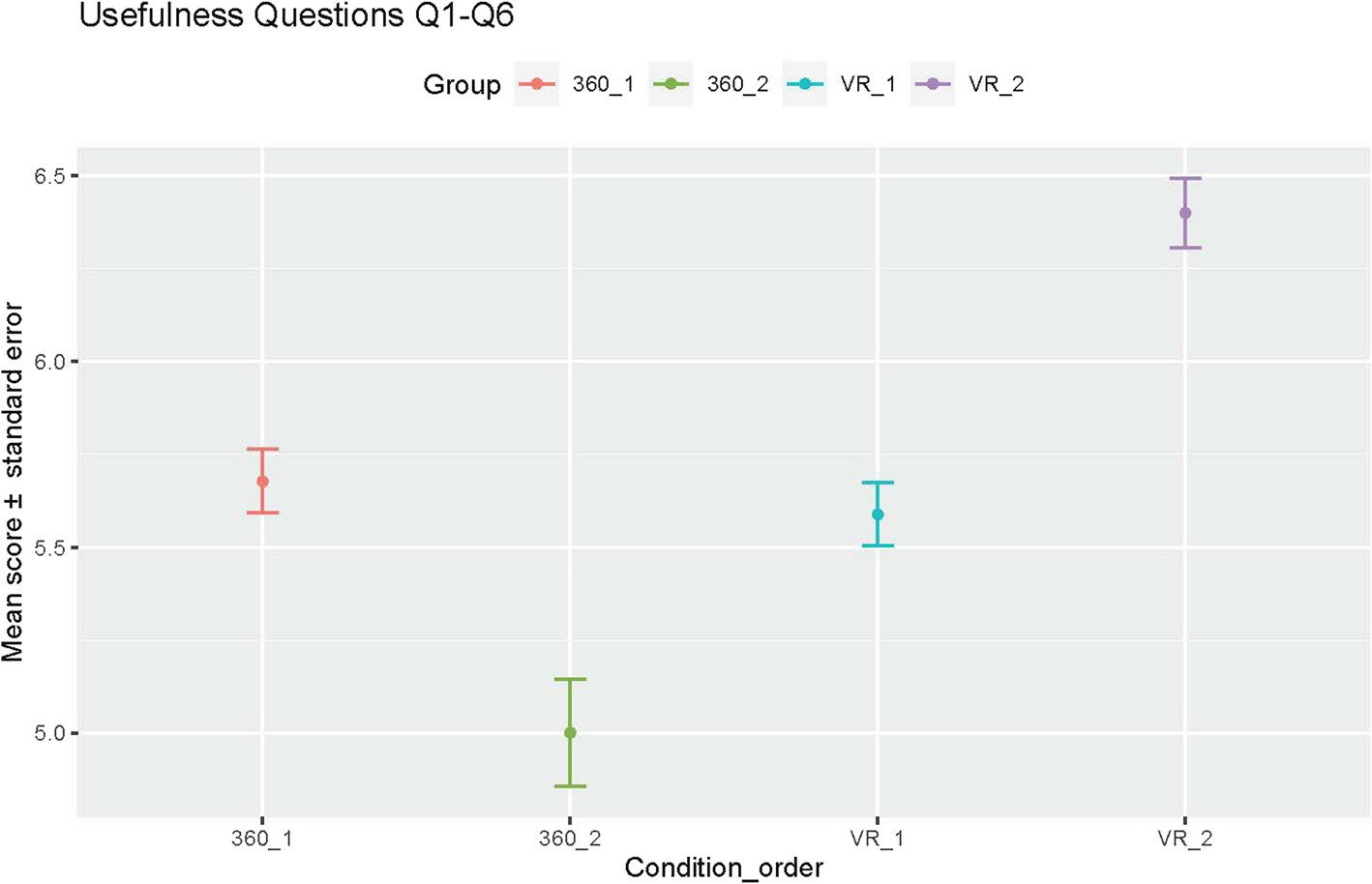
Mean score and standard error for the perception of usefulness when grouping by condition and order

Participants found both conditions easy to use, with mean rankings of 6.03 ± 1.13 and 6.13 ± 0.94 across all questions for 360° video and VR, respectively. For all group pairings, no statistically significant differences were found in participants’ ratings of “ease of use”. When “usefulness” was compared, there was a significant difference in the perception of usefulness regardless of order (mean usefulness score was 5.3 ± 1.3 for 360° video and 6.0 ± 0.86 for VR). The difference in usefulness ratings was negligible between groups 360_1 vs VR_1 and quite prominent in favor of VR between groups 360_2 vs VR_2 (mean usefulness score was 5.0 ± 1.37 for 360_2 and 6.4 ± 0.9 for VR_2, Fig. 5), suggesting usefulness perceptions became stronger following exposure to both conditions (e.g. 360° video and VR). Similarly, when usefulness was compared between groups VR_1 vs. VR_2, all questions showed a significant difference in favor of VR_2 (median of 6 with an interquartile range of 1 vs median of 7 with an interquartile range of 1, Fig. 5). The results from an ANOVA analysis suggest that the most significant factor in the participants’ usefulness scores is the condition (F statistic 38.9, *p*-value < 1.3 × 10^−9^), the order is not a significant factor (F statistic 0.4, *p*-value 0.53), and there is a significant interaction effect between condition and order (F statistic 50.16, *p*-value 7.8 × 10^−12^). This suggests that exposure to both conditions impacts the perception of the usefulness of both conditions among participants.

Most participants reported no symptoms of simulator-induced sickness with only some reporting slight and moderate symptoms for some questions. Both study groups also performed well in the PPV demonstration following exposure to their initial condition, with a median total score of 12/15 and an interquartile range of 2.75. There was no overall significant difference in instructor-observed scores for the 360° video or VR simulation conditions. However, when reviewing specific observation checklist criteria, participants completing the VR simulation did better on Q7: “Provides effective PPV (40–60 bpm) for 30 s” (mean score 1.2 ± 0.56 vs 1.7 ± 0.46, adjusted *p*-value 0.005) and Q8: “Re-evaluates heart rate (Heart rate must be greater than 100 bpm)”, than participants watching the 360° video (mean score 1.7 ± 0.49 vs 1.9 ± 0.26, adjusted *p*-value 0.04).

## Discussion

In this study, we evaluated the effects of 360° video vs VR simulation on NRP provider satisfaction, perceptions of presence and confidence in performing neonatal resuscitation, and ability to perform positive pressure ventilation. NRP providers found both conditions useful and showed an increase in their self-reported confidence in PPV after using either 360° video or VR simulation. However, there was a clear statistical difference in the perception of usefulness and presence, with participants reporting the VR simulation to be more helpful in improving their learning performance, enhancing their learning and an increased sense of presence, suggesting a greater perception of immersion and involvement. While participants in both VR and 360° video study groups performed well in the PPV demonstration, those exposed initially to the VR simulation demonstrated better performance in providing effective PPV and re-evaluating heart rate.

According to Yeo et al. [36], immersive VR applications using handheld controllers seem promising for use in NRP, but research on their effectiveness is limited. Garvey and Dempsey [7] suggest that VR creates realistic scenarios involving all senses that are reproducible and without instructor variation, allowing for trainees to review and potentially practice resuscitation skills in their own time, and reducing the need for instructors and manikin-based simulation. This study contributes to the limited literature surrounding the use of extended reality technologies in NRP. It is novel in that it compares using different extended reality types (360° video and VR) across various learning outcomes. The evaluation of the effect of extended reality system use on positive pressure ventilation performance has also not been examined in previous studies. An extensive description of the experimental setup, and a detailed breakdown of the results and further analysis is available in the work of Aydin [37].

Immersion and presence have been identified as two key features of VR technology, with a higher degree of perceived immersion influencing a user’s subjective experience of presence [27]. Examining users’ perception of “presence” is a unique aspect of the study that has not been investigated extensively. Presence has been identified as a key factor in immersive learning experiences and a key feature of VR that is conducive to learning. Immersive VR systems and simulations have the potential to enhance learner engagement, increase satisfaction, and foster greater contextualization for users. Experiences of immersion have also been shown to be more significant when extended reality simulations afford more interaction and engagement within a virtual environment [14]. VR simulations, in comparison to 360° video, have the advantage of re-creating realistic environments that can involve multiple senses and invoke a psychological state in which the user feels as though they are interacting directly within a simulated setting. Creating that sense of presence through immersion is the primary goal of using immersive VR in education and training. Some authors have also suggested that immersive VR may add more value when compared to traditional manikin simulation sessions due to the increased level of situational stress created by interaction in the virtual environment [14]. Our previous work evaluating 360° video systems alone for neonatal resuscitation training found that participants were interested in virtual simulations that involved greater interactivity [2]. The findings from our study suggest a higher level of presence and immersion experiences amongst users of the VR simulation when used initially, with this subjective experience towards VR becoming even more pronounced following the use of both VR and 360° video systems.

As a mode of simulation-based education, extended reality technologies empower healthcare providers to experience simulation scenarios at their own pace and observe unfamiliar or rare scenarios [2]. This technology may provide a way to supplement traditional instructional methods in NRP training. Since rural healthcare providers may experience less exposure to neonatal emergencies and fewer opportunities to undertake formal booster or refresher training [38], extended reality systems could enhance access for providers to boost resuscitation knowledge. Regularly scheduled booster or refresher updates using manikin simulators require equipment and space, as well as coordinating schedules of both instructor and staff. Extended reality could provide greater access to standardized and portable refresher learning opportunities on NRP concepts, and offer some longer-term cost-efficiencies as a modality for simulation-based education [2].

The limitations of our study were the small sample size and the wide diversity in the healthcare provider backgrounds of our sample, which could limit generalizability. Most participants had also completed NRP in the last year, so their NRP knowledge and skills may have been more up-to-date, which may have minimized the opportunity for observing greater outcome gains. There may have also been technical limitations in the conditions that influenced participants’ experiences. Lastly, another study limitation was the mandatory use of masks due to COVID-19 pandemic regulations. Some participants reported foggy glasses, which may be related to mask-wearing.

## Conclusions

The study findings suggest a high level of acceptance of VR headsets and 360° video amongst healthcare providers trained in NRP. NRP providers found both systems useful; however, VR simulation was more helpful in improving learning performance, enhancing learning, and providing an increased feeling of presence. Participants reported an increased feeling of confidence in their own neonatal resuscitation skills after exposure to VR and performed better on a crucial NRP skill, providing effective PPV. The findings also support potential opportunities for adopting VR headsets as a modality for refreshing resuscitation knowledge and skills. It enables learners to experience immersion in resuscitation scenarios without being present in real clinical settings and provides them with valuable self-learning and supplementary learning opportunities when there are limited in-person training opportunities.

## Data Availability

The datasets used and/or analysed during the current study are available from author LPC on reasonable request.
